# Widely Targeted Metabolomics Analysis Reveals Metabolites Important for Antioxidant Properties and Quality Traits in Different Fruit Parts of Aurantii Fructus Immatures

**DOI:** 10.3390/molecules29081733

**Published:** 2024-04-11

**Authors:** Shuo Zhang, Ze Liu, Xinyu Xu, Ruihua Zhao, Shujiang Zhang, Rong Luo

**Affiliations:** School of Traditional Chinese Medicine, Capital Medical University, Beijing 100069, China; zsboluo@mail.ccmu.edu.cn (S.Z.); 1416544635@mail.ccmu.edu.cn (Z.L.); 2280769290@mail.ccmu.edu.cn (X.X.); 13237487989@mail.ccmu.edu.cn (R.Z.); zhangshujiang2020@mail.ccmu.edu.cn (S.Z.)

**Keywords:** Aurantii Fructus Immatures, quality evaluation, metabolic profiling, antioxidant activity, chemometrics

## Abstract

In traditional Chinese medicine, Aurantii Fructus Immatures (AFIs) have been utilized for more than 2000 years. The proportions of different fruit parts are crucial for evaluating AFI quality in China. However, the basis for this statement’s substance is unclear. Differences in quality are intimately correlated with a plant’s metabolite composition. On the basis of a widely targeted metabolome, this study intended to investigate the metabolite composition and evaluate the antioxidant capacity of the peel and pulp of an AFI. Metabolites were identified and quantified by UHPLC-QqQ-MS. To assess their antioxidant ability, DPPH and ABTS assays were carried out. There were 1327 chemical compounds identified by UHPLC-QqQ-MS. After screening the differential metabolites using a multivariate statistical analysis, it was found that there were 695 significant differences in the metabolites between the peel and the pulp. Among them, it was discovered that the content of active ingredients in the peel group was higher than that in the pulp group. Furthermore, the aqueous extracts from the peel showed stronger antioxidant capacities than those from the pulp. The metabolites and antioxidant capacities were significantly different between the peel and the pulp. This study of different fruit parts might provide a guide for AFI quality assessments.

## 1. Introduction

Citrus fruits are highly popular worldwide [1]. Typically, mature citrus fruits are used to make a variety of foods, including marmalade, drinks, and oils [2]. Variations in the compositions of citrus fruits at different ripening stages provide them with additional properties and applications, while the immature fruits of some citrus varieties are preferred by people due to their medicinal properties. The most well-liked of these are Aurantii Fructus Immatures (AFIs; Zhishi in Chinese), which have been utilized extensively as Chinese herbal medicines (CHMs). AFIs are the dried immature fruits of *Citrus aurantium* L. and its cultivars or *Citrus sinensis* Osbeck [3,4]. They are distributed all over the world, especially in the southern regions of China, and are credited with regulating qi and aiding digestion [5].

Currently, AFIs are receiving more attention due to their health-promoting effects and bioactivities [6]. Modern phytochemical research has found that AFIs contain various bioactive substances, particularly flavonoids, which act as free radical scavengers and display strong antioxidant capabilities [1,4]. Nevertheless, the types and contents of these bioactive substances in AFIs have not been sufficiently investigated. The bioactivities and health beneficial properties of AFIs include anticancer [7], antianxiety [8], anti-obesity [9,10], antibacterial [11], and antioxidant [12] capabilities. As a result, AFIs are used to treat a variety of conditions, including gastrointestinal disorders, anxiety, and lung cancer [6,7,8,9,10,11,13,14].

It is well recognized that the qualities and therapeutic efficacies of CHMs are intimately correlated with their bioactive components, which are primarily secondary metabolites accumulating in different tissues or parts of the CHMs [15,16,17]. The *Illustrated Classic of Material Medica* states the following: “AFI with a higher proportion of peel thickness are more efficacious”. Until now, traditional Chinese pharmacists and farmers have also believed that AFIs with lower proportions of pulp thickness will have better quality. Although the effects of different fruit parts’ thicknesses on their efficacies are documented in medical classics and clinical applications, the basis for this statement’s substance is unclear. Interestingly, modern studies have suggested that the distributions of secondary metabolites are plant-part specific [18,19,20]. However, previous studies on AFIs have frequently concentrated on whole fruits. Moreover, concerning differences in the metabolomic compositions of different fruit parts of AFIs, very limited information is available.

In recent years, widely targeted metabolomics analyses, which primarily use ultrahigh performance liquid chromatography coupled with triple quadrupole mass spectrometry (UHPLC-QqQ-MS) techniques, have emerged as a potent method that combines the benefits of both non-targeted and targeted metabolomics [21]. Due to its characteristics of quick separation, high sensitivity, broad coverage, and high throughput, this approach has been used extensively in plant metabolite analyses for many herbs [22,23,24]. Thus, it can serve as a useful method for performing qualitative and quantitative analyses to quickly identify a variety of metabolites in AFIs. In view of this, this study aimed to investigate the metabolite compositions and variations in the peels and pulps of AFIs using widely targeted metabolomics and chemometrics. At the same time, this approach was combined with an antioxidant ability experiment to compare the antioxidant capacities of the peels and pulps of AFIs. These results will serve as a foundation for further quality evaluations of AFIs.

## 2. Results

### 2.1. Metabolomic Profiling

**Figure 1:**
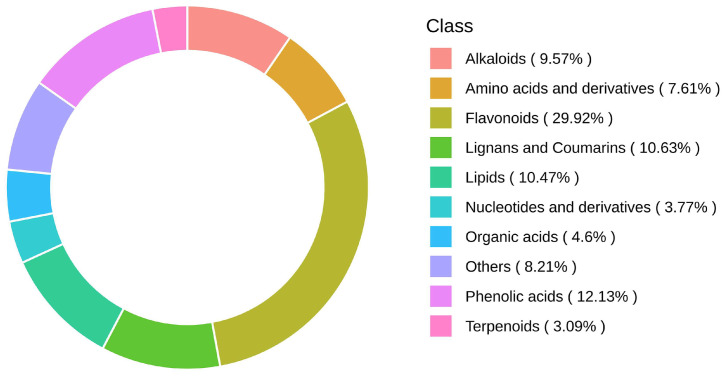
AFI metabolite composition ring diagram.

Total ion current (TIC) plots and an extracted ion chromatogram (XIC) plot in MRM mode of one QC sample are shown in Figure S1 (Supplementary Materials). A total of 1327 compounds were identified, including 397 flavonoids, 161 phenolic acids, 141 lignans and coumarins, 139 lipids, 127 alkaloids, 101 amino acids and derivatives, 61 organic acids, 50 nucleotides and derivatives, 41 terpenoids, and 109 other metabolites (Figure 1). Detailed data on all the identified metabolites are shown in Table S1 (Supplementary Materials).

An overlay analysis was used to evaluate the technical repeatability of the metabolite extractions and detections. In Figure S2A,B (Supplementary Materials), an overlay of the TIC plots for three QC samples is shown, indicating that the TIC plots of the metabolites had a perfect overlap, that is, the retention time and peak intensity were consistent, demonstrating good sample homogeneity.

### 2.2. PCA and OPLS-DA for the Peel and Pulp of AFIs

**Figure 2:**
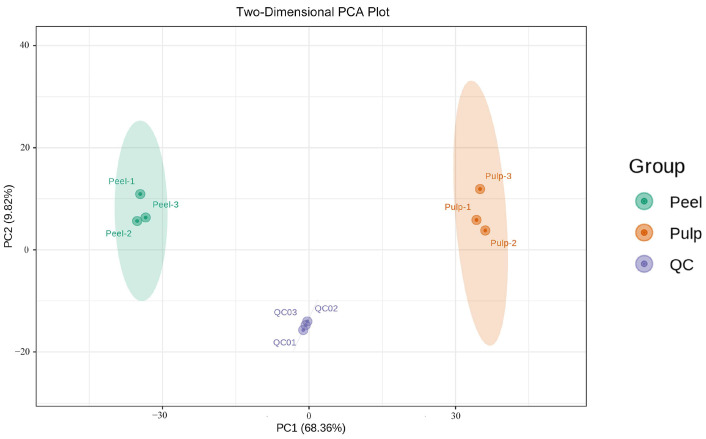
PCA plot from the peel and pulp groups. The samples in the same group are clustered into a cluster, and the ellipse represents the 95% confidence interval.

**Figure 3:**
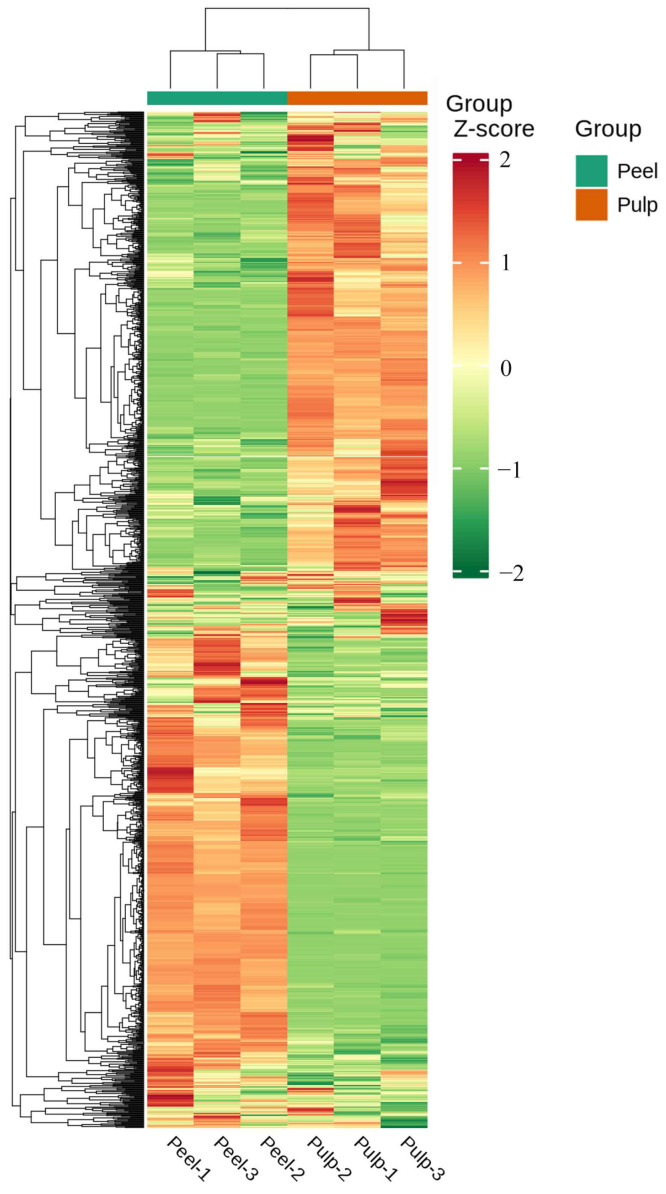
Heatmap of the groups of peel and pulp.

In order to compare the metabolite compositions of the peels and pulps, a PCA analysis was performed on the dataset obtained by UHPLC-QqQ-MS/MS in ESI^+^/ESI^−^ mode. As shown in Figure 2, two principal components (PC1 and PC2) contributed 68.36% and 9.82% of the variation, respectively. The results show that the two groups were obviously separated, and the three biological replicates of each variety were closely clustered together, indicating that the experiment had good repeatability and reliable results. This comparison showed a significant difference between the peel and pulp, and all samples fell within the 95% confidence interval. Furthermore, they were clearly divided into two categories on the heatmap (Figure 3), indicating significant differences in metabolite content between the peel and the pulp. These results demonstrate the differences in metabolites between the two different fruit parts of AFIs.

**Figure 4:**
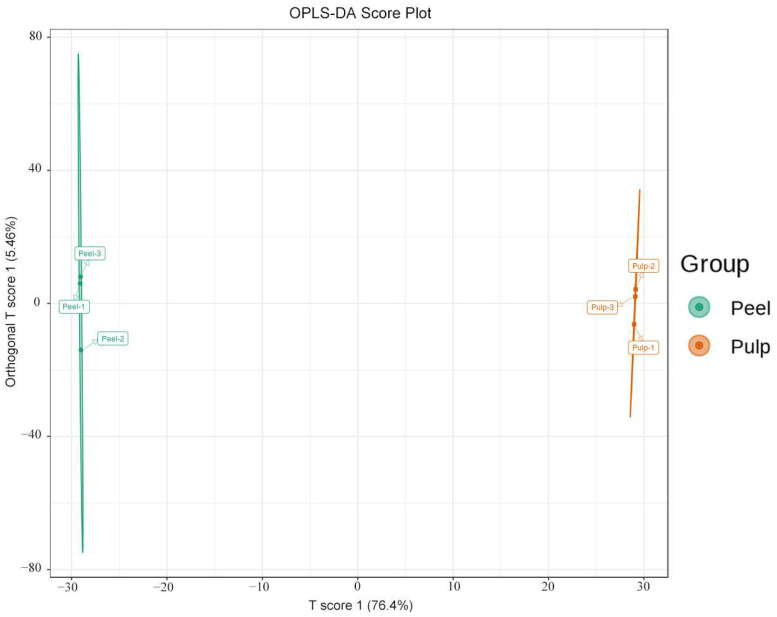
Plot of OPLS-DA scores for the groups of peel and pulp.

**Figure 5:**
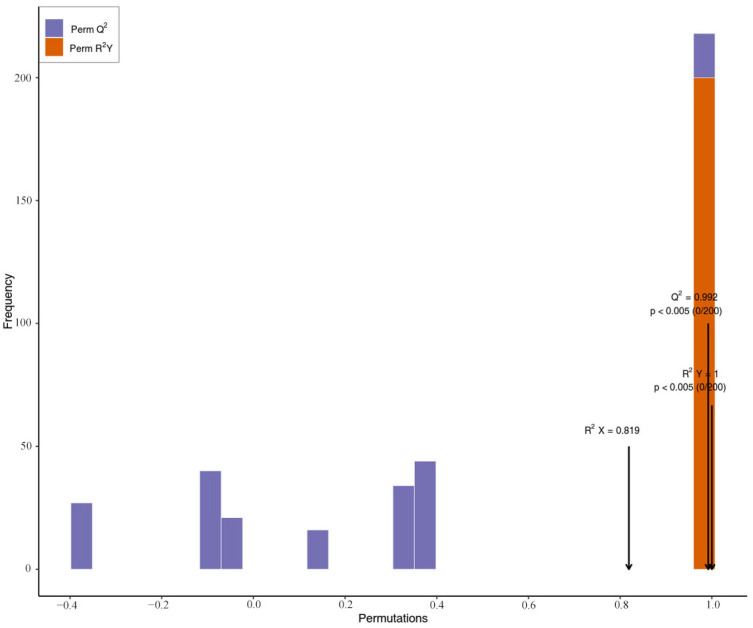
OPLS-DA model simulation verification diagram for the groups of peel and pulp.

To further screen the variables responsible for the peel and pulp of AFIs, a supervised OPLS-DA model was subsequently established. As seen in Figure 4, samples from the two groups were significantly and accurately segregated into two different parts, suggesting significant differences in the metabolites of the two parts, and the outcome was similar to the PCA results. The OPLS-DA model (Figure 5) compared the metabolite contents of the different fruit parts in pairs to evaluate differences between the peel and the pulp (R^2^X = 0. 819; R^2^Y = 1; Q^2^ = 0.992). The Q^2^ values in this study were greater than 0.9, indicating the excellent fitness and predictability of the model. The above results revealed that the established OPLS-DA model was reliable and could be used to screen the variables responsible for the peel and the pulp of AFIs.

### 2.3. Differential Metabolite Screening, Functional Annotation, and Enrichment Analysis between Peel and Pulp

**Figure 6:**
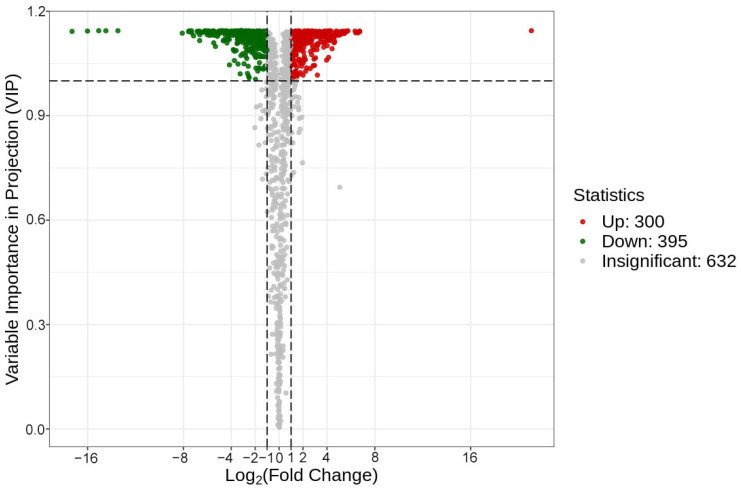
Volcano plots of differential metabolites. The horizontal dotted line represents the threshold of 1 for VIP. The vertical dashed lines represent the thresholds of −1 and 1 for log_2_ (fold change), respectively (thresholds 0.5 and 2 for fold change).

We further conducted a differential metabolite screening based on the fold change (FC ≥ 2 or FC ≤ 0.5) and variables identified as necessary in the projection scores (VIP ≥ 1) (Table S2 (Supplementary Materials)). The results of the screening were presented in the form of a volcano plot (Figure 6). There were 695 significantly different metabolites between the peel and the pulp. Among them, compared with the pulp, 300 metabolites in the peel were upregulated, that is, the relative content was increased, and 395 metabolites were downregulated, that is, the relative content was decreased. The most significant difference was found for flavonoids, accounting for about 34.68% (Table 1). It is worth noting that almost all polymethoxylated flavones (PMFs) were downregulated, indicating that the relative content of PMFs in the peel was significantly higher than that in the pulp. Numerous studies have recently reported the powerful biological activities of PMFs, which play an important role in antioxidant, antibacterial, anticancer, antiobesity, antidiabetic, etc., activities [25]. These results show that secondary metabolites vary between the peel and the pulp of AFIs.

**Figure 7:**
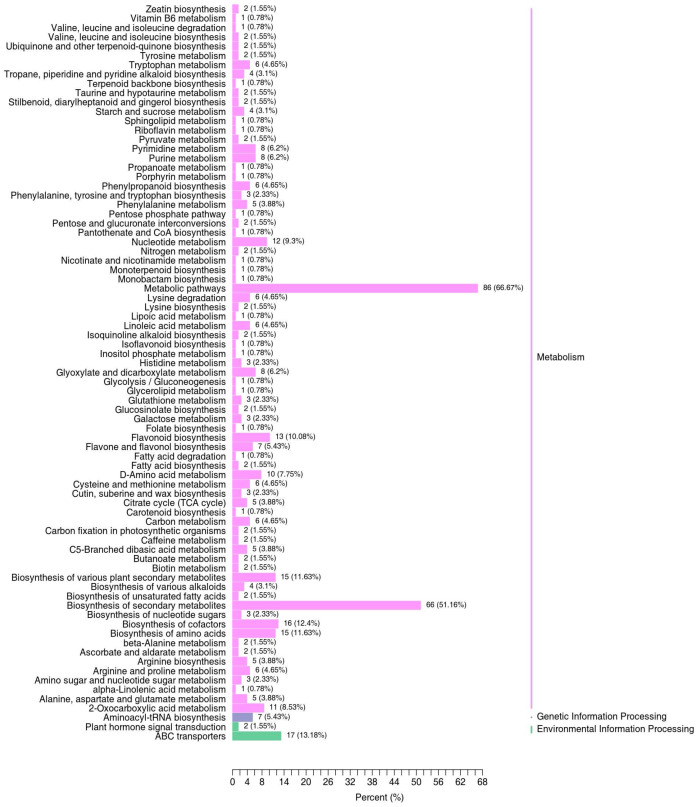
KEGG classification of differential metabolites.

**Figure 8:**
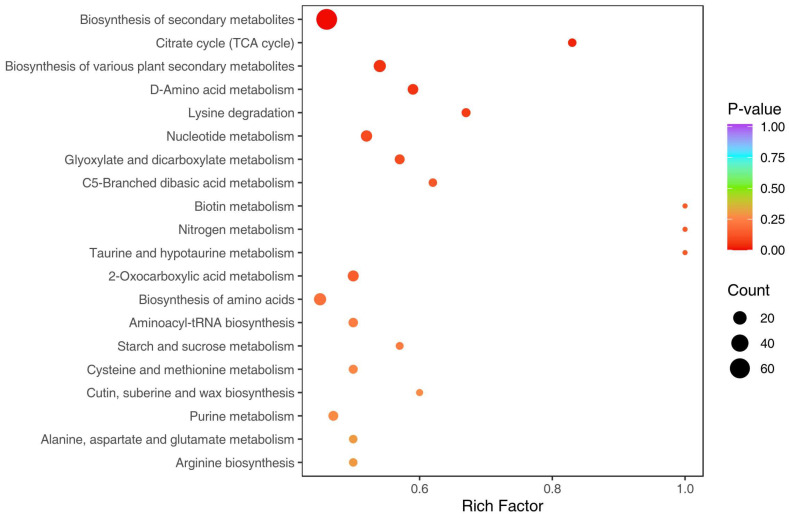
Differential metabolite pathway bubble map.

A pathway annotation and an enrichment analysis using the KEGG pathway database were performed for the vital differential metabolites. The differential metabolites were involved in 79 pathways (Figure 7, Table S3 (Supplementary Materials)). The results showed that the top 10 pathways with the highest numbers of differential metabolites were metabolic pathways (86), the biosynthesis of secondary metabolites (66), ABC transporters (17), the biosynthesis of cofactors (16), the biosynthesis of various plant secondary metabolites (15), the biosynthesis of amino acids (15), flavonoid biosynthesis (13), nucleotide metabolism (12), 2-Oxocarboxylic acid metabolism (11) and D-Amino acid metabolism (10). In addition, the top 20 significant pathways are presented in bubble plots (Figure 8, Table S4 (Supplementary Materials)). A metabolic pathway analysis of differential metabolites screened in the peel and pulp indicated that two pathways with *p* < 0.05 in the enrichment analysis were the biosynthesis of secondary metabolites and the citrate cycle. These results suggest that the effects of different fruit parts’ thicknesses on efficacies may be explained by the metabolic pathway, mainly due to differences in the biosynthesis of secondary metabolites, especially flavonoids.

### 2.4. Antioxidant Activities of Different Fruit Parts in AFIs

In this investigation, DPPH and ABTS scavenging activity assays were used to preliminarily analyze the antioxidant activities of aqueous extracts from the peel and pulp. Antioxidant capacities were indicated by an IC_50_ value: the lower the IC_50_ value, the greater the antioxidant capacity. The findings demonstrated that the IC_50_ in the DPPH assay of the peel was lower than that of the pulp (Table 2). Furthermore, there was a significant difference in the IC_50_ in the DPPH assay between the peel and the pulp.

As shown in Table 2, the ABTS antioxidant capacities of the aqueous extracts from the peel were also higher than those of the pulp, with IC_50_ values of 0.130 ± 0.08 mg·mL^−1^ and 0.211 ± 0.010 mg·mL^−1^, respectively. Similar to the results of the DPPH assay, the difference in the ABTS antioxidant capacities between the peel and the pulp was significant.

## 3. Discussion

The proportions of different fruit parts of AFIs are significant in traditional Chinese medicine. Since the time of records of herbalism from the Song Dynasty, traditional Chinese pharmacists and farmers have been paying attention to the different characteristics of the peel and pulp of AFIs. They noted that a higher proportion of peel thickness is an essential characteristic of high quality in AFIs. This traditional quality assessment method is consistent with the current research results on citrus, which indicate that the distribution of some metabolites in citrus fruits has tissue specificity [26]. However, this needs to be further clarified in AFIs. Only a few types of metabolites between the peel and the pulp of AFIs have been studied thus far. These compounds include four flavonoids (narirutin, naringin, hesperidin, and nobiletin) and one alkaloid (Synephrine) [27]. In the present study, we performed a widely targeted metabolomics analysis for the peel and the pulp of AFIs and provided a partial metabolic profile of this popular functional food.

Based on the findings of the metabolomics analysis, 1327 chemical compounds were discovered from the extracts, including a significant number of phytochemicals. There were 161 phenolic acids in the AFIs in total. Some phenolic acids, such as caffeic acid, ferulic acid, vanillic acid, and gallic acid, were detected at significantly high levels. According to the current findings, most of these chemicals were also previously documented in extracts of AFIs [4,28,29]. These highly accumulated compounds have been shown to possess beneficial bioactivities. For example, caffeic acid is a phenolic acid and a catechol derivative possessing anticancer, anti-AIDS, antioxidant, and anti-inflammatory activities [30]. Ferulic acid has attracted attention for its potential role as an adjuvant therapy for several free-radical-induced diseases [31]. Vanillic acid promotes favorable outcomes in various disease models due to its potent antioxidant and antimicrobial properties [32]. In addition, 141 lignans and coumarins, 139 lipids, 127 alkaloids, 101 amino acids and derivatives, 61 organic acids, 50 nucleotides and derivatives, 41 terpenoids, and 109 other metabolites were also identified in the AFIs.

We performed a statistical analysis of the metabolites, and significant differences were found between the peel and the pulp. A metabolic pathway analysis of the differential metabolites in the peel and the pulp revealed that flavonoid biosynthesis contained a high number of differential metabolites, indicating significant differences in the relative content of flavonoids between the peel and the pulp. It is noteworthy that 61 of the 241 differential flavonoid compounds were PMFs, that is, a chemical family of flavones with a number of methoxyl groups equal to or greater than four [33]; moreover, these PMFs were significantly higher in the peel than in the pulp. Functionally, PMFs have received considerable attention as strong antioxidant compounds with various bioactivities as anti-atherosclerosis, anti-inflammation, neuroprotection, anti-cancer, and anti-microbial activities [34]. Thus, enhanced antioxidant activities in the peel of AFIs may be correlated with an increase in the relative levels of PMFs. Similar to the results in the present study, Helena et al. [35] found that the in vitro antioxidant capacities of all peels were higher than those of pulps in four Citrus species (*C. sinensis*, cvs. Pera and Lima; *C. latifolia* Tanaka cv. Tahiti; *C. limettioides* Tanaka cv. Sweet lime and *C. reticulate*, cv. Ponkan), which was associated with high PMF contents. With the help of earlier research, we surmise that the significant differences in the content of flavonoids, particularly PMFs, are associated with clinical effects.

A pharmacological study indicated that PMFs isolated from citrus peels could improve impaired intestinal barrier function caused by epithelial damage and the dysregulation of tight junction proteins, modulating the gut microbiome toward a healthier profile [36]. In our study, the presence of more PMF metabolites, such as 5-Demethylnobiletin, Isosinensetin, 3,5,6,7,8,3′,4′-Heptamethoxyflavone, and 6-Demethoxytangeretin, in the peel than in the pulp, tended to strengthen the regulating qi and aid the digestive effects of AFIs. AFIs’ pharmacological action and therapeutic effectiveness may differ depending on the types and quantities of these pharmacologically active metabolites.

The clinical effectiveness of traditional Chinese medicine, particularly its active ingredients, is impacted by the intricate chemical makeup of medicinal plants [37]. The difference in metabolites between the peel and the pulp resulted in the quality of the AFIs, affecting clinical outcomes. This research via metabolomics analysis may provide a partial scientific basis for the quality evaluation of AFIs. The correlation between the types or contents of components in the peel and pulp of AFIs and AFIs’ qualities or efficacy still needs further verification, such as the impact of different varieties and habitats.

## 4. Materials and Methods

### 4.1. Plant Materials

**Figure 9:**
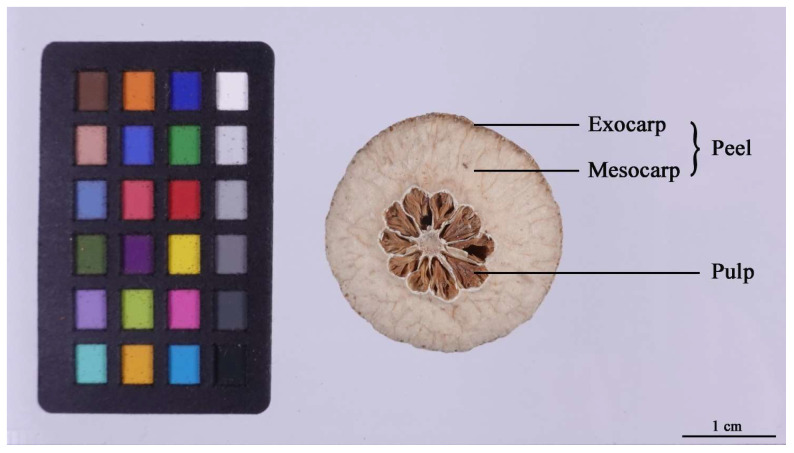
A structure diagram of an Aurantii Fructus Immature (AFI) with a standard color checker.

The study materials for the widely targeted metabolomics analysis consisted of immature fruits of *Citrus aurantium* L., which were obtained from Liugongmiao Town, Zhangshu City, Jiangxi Province, China, in June 2022. In addition, the study materials for the determination of antioxidant capacities included 12 groups of immature fruits of *Citrus aurantium* L. which were bought from a traditional Chinese medicine market in Jiangxi Province, China. The pulp was gently peeled off using tip tweezers to separate the pulp from the peel (Figure 9). The samples were packed in polythene pouches and stored at 20 °C in a pharmaceutical cooling cabinet until further analysis.

### 4.2. Reagents

Liquid chromatography-grade acetonitrile and methanol were acquired from Merck (Darmstadt, Germany). Aladdin (Shanghai, China) provided the formic acid and potassium persulfate. 2,2-diphenyl-1-picrylhydrazyl (DPPH) and 2-azino-bis-(3-ethylbenzothiazoline-6-sulphonic acid) diammonium salt (ABTS) were obtained from Macklin (Shanghai, China).

### 4.3. Widely Targeted Metabolomics Analysis

#### 4.3.1. Sample Preparation and Extraction

The peel and pulp samples were vacuum freeze-dried in a lyophilizer (Scientz-100F; SCIENTZ, Ningbo, China), and they were then ground into a fine powder using a grinder (MW400; Retsh, Haan, Germany) at 30 HZ for 1.5 min. Then, 50 mg of powder was mixed with 1.2 mL of 70% aqueous methanol, vortexed 6 times for 30 s every 30 min, and left at 4 °C overnight. The supernatant was separated and filtered using microporous membranes (0.22 μm pore size, ANPEL, Shanghai, China) and then stored in vials following centrifugation at 12,000 rpm for 10 min. The peel and pulp samples were divided into two groups; each sample had three biological replicates. Quality control (QC) samples were prepared by mixing extracts and inserting one QC sample for every two samples analyzed [21,38,39].

#### 4.3.2. UHPLC Conditions and ESI-Q TRAP-MS/MS

The extracts were analyzed using a UPLC-ESI-MS/MS system (UPLC, ExionLC™ AD; MS, Applied Biosystems 4500 Q TRAP, AB Sciex, Foster City, CA, USA). The analytical parameters were as follows: UPLC—an Agilent SB-C18 (1.8 μm, 2.1 mm × 100 mm, Agilent, Santa Clara, CA, USA) chromatographic column was applied. The mobile phase consisted of solvent A (pure water containing 0.1% formic acid) and solvent B (acetonitrile containing 0.1% formic acid). The samples were determined using a gradient program with a starting condition of 95% solvent A and 5% solvent B. A linear gradient to 5% solvent A and 95% solvent B was conducted within 9 min and kept in this state for 1 min. Within 1.1 min, a composition of 95% solvent A and 5.0% solvent B was programmed and kept for 2.9 min. The column oven was set to 40 °C. The flow velocity was set at 0.35 mL per minute. The injection volume was 4 μL, and the effluent was alternatively connected to an ESI–triple quadrupole–linear ion trap (Q TRAP) MS system [21,24,39].

The triple quadrupole–linear ion trap mass spectrometer (Q TRAP) API 4500 Q TRAP UHPLC/MS/MS System, equipped with an ESI Turbo Ion-Spray interface, operated in positive and negative ion modes and was controlled by Analyst 1.6.3 software (AB Sciex, Foster City, CA, USA). The ESI source operation conditions were as follows: ion source—turbo spray; source temperature—550 °C; ion spray voltage (IS)—5500 V (positive ion mode)/—4500 V (negative ion mode); and ion source—gas I (GSI), gas II (GSII), and curtain gas (CUR), which were set at 50, 60, and 25 psi, respectively; the collision-activated dissociation (CAD) was high. QqQ scans were acquired as MRM experiments with the collision gas (nitrogen) set to medium [21,37,39].

#### 4.3.3. Analyzing Metabolites Qualitatively and Quantitatively

Based on the self-built database MWDB (MetWare Biological Science and Technology Co., Ltd., Wuhan, China), primary and secondary mass spectrometry data were analyzed qualitatively. During the data analysis, repetitive signals made up of NH^4+^, Na^+^, and K^+^, as well as isotope signals and fragments of other compounds with a higher molecular weight, were eliminated [39].

Utilizing triple quadrupole mass spectrometry, metabolites were analyzed quantitatively using the multiple-reaction-monitoring (MRM) mode. In the MRM mode, QqQs were used to select single-fragment ions with the desired characteristics. All chromatographic peaks were submitted to area integration and correction by MultiQuantv 3.0.2 (AB Sciex, Concord, ON, Canada) after the metabolite mass spectrometry data were collected for each sample. Chromatographic peak area integrals expressed the corresponding relative metabolite contents [37,39].

#### 4.3.4. Multivariate Statistical Analysis

In order to achieve data standardization and homogenous variance, the metabolite data were log-transformed to normalize them. Metabolites from the two groups of samples were used for a principal component analysis (PCA) within R (base package 3.5.1), a cluster analysis with R (ComplexHeatmap 2.8.0), and an orthogonal partial least squares discriminate analysis (OPLS-DA) within R (MetaboAnalystR 1.0.1). Differential metabolites were annotated using the Kyoto Encyclopedia of Genes and Genomes (KEGG) compound database (http://www.kegg.jp/kegg/compound/; accessed on 21 October 2022), and the annotated metabolites were then mapped to the KEGG pathway database (http://www.kegg.jp/kegg/pathway.html; accessed on 21 October 2022).

### 4.4. Determination of Antioxidant Capacities

#### 4.4.1. Sample Preparation and Extraction

The peel and pulp samples were ground into a powder and sieved through a 65-mesh sieve, respectively. Then, the extracts were extracted by carrying out a refluxing extraction with 0.5 g of the powders and 10 mL of deionized water at 100 °C for 1 h. The filtered residue was refluxed and extracted for 1 h as before. After combining and concentrating the filtrate, the enriched solution was diluted to a suitable concentration.

#### 4.4.2. DPPH Assay

The DPPH assay followed the method described by Guo et al., with some modifications [21,40,41]. That is, 0.1 mL of a 0.2 mM DPPH^•^ methanolic solution as mixed with 0.1 mL of extracted solutions at varying concentrations (0.001~0.9 mg·mL^−1^), which were provided in microtubes. The mixture was automatically shaken, and the absorbance was measured at 517 nm (SpectraMax^®^ iD3, Molecular Devices, San Jose, CA, USA) immediately after incubation at 25 °C for 30 min. Vitamin C (V_C_) was employed as a positive control. The DPPH radical scavenging capacity was calculated using the following formula:

(1) $${\mathrm{Scavenging}\;\mathrm{rate}}_{\left(\mathrm{DPPH}\right)}\%=1-\frac{\left(A_{\mathrm{sample}}-A_{\mathrm{control}}\right)}{A_{\mathrm{blank}}}$$

where *A_sample_* is the absorbance of the DPPH^•^ methanolic solution with the extracted solution, *A_control_* is the absorbance of the methanolic solution with the extracted solution, and *A_blank_* is the absorbance of the DPPH^•^ methanolic solution with deionized water. The concentration required for the half-maximal inhibitory concentration (IC_50_) was used to represent the antioxidant activities using GraphPad Prism 8.0.2 software (GraphPad, San Diego, CA, USA).

#### 4.4.3. ABTS Assay

The ABTS assay followed the method described by Opitz et al., with some modifications [40,41,42]. That is, 2.5 mM potassium persulphate was added to a 7.4 mM ABTS aqueous solution (1:1, *v*/*v*), and the reaction was carried out at 25 °C for 12 h under light-protected conditions. The ABTS^•+^ solution was diluted with absolute methanol to an absorbance of 0.70 ± 0.02 at 734 nm to obtain an ABTS^•+^ radical working solution. Then, 0.1 mL sample extracts with varying concentrations (0.001~0.6 mg·mL^−1^) were added to the ABTS^•+^ solution in a microplate. The mixture was automatically shaken, and the absorbance was measured at 734 nm immediately after incubating it at 25 °C for 6 min. V_C_ was employed as a positive control. The ABTS radical scavenging ability was calculated using the following formula:

That is, 2.5 mM aqueous potassium persulfate (1:1, *v*/*v*) was added to a 7.4 mM aqueous ABTS solution, and the reaction was carried out at 25 °C for 12 h under light-protected conditions.

(2) $${\mathrm{Scavenging}\;\mathrm{rate}}_{\left(\mathrm{ABTS}\right)}\%=1-\frac{\left(A_{\mathrm{sample}}-A_{\mathrm{control}}\right)}{A_{\mathrm{blank}}}$$

where *A_sample_* is the absorbance of the ABTS^•+^ solution with the extracted solution, *A_control_* is the absorbance of the methanolic solution with the extracted solution, and *A_blank_* is the absorbance of the ABTS^•+^ methanolic solution with deionized water. The concentration required to reach the IC_50_ was used to represent the antioxidant activities using GraphPad Prism 8.0.2 software.

## 5. Conclusions

In this study, a UHPLC-QqQ-MS/MS-based metabolomics approach was used to evaluate differences in metabolites between the peel and pulp of AFIs. This is the first report of metabolomics in AFIs, and a total of 1327 metabolites in *Citrus aurantium* L. were identified. In total, 695 significantly different metabolites were identified by comparing the peel and the pulp. A metabolic pathway analysis of the differential metabolites revealed that the biosynthesis of secondary metabolites was significantly enriched, and flavonoid biosynthesis demonstrated more differential metabolites. Furthermore, the aqueous extracts from the peel showed stronger antioxidant capacities than those from the pulp.

In summary, based on the quality evaluation, which included chemical aspects and biological effects, we found that the peel had higher levels of some active ingredients and increased antioxidant activities in comparison with the pulp. Therefore, the scientific connotation of the traditional quality evaluation standard in which “AFIs with a higher proportion of peel thickness are more efficacious” was preliminarily confirmed. The study of different fruit parts might provide a guide for AFI quality assessments.

## Figures and Tables

**Table 1:** Classification and statistics of metabolites with different relative contents between the peel and pulp of AFIs.

Type	Number	Percentage
Flavonoids	241	34.7
Lignans and coumarins	114	16.4
Phenolic acids	77	11.1
Lipids	75	10.8
Alkaloids	63	9.1
Others	32	4.6
Amino acids and derivatives	30	4.3
Organic acids	23	3.3
Terpenoids	22	3.2
Nucleotides and derivatives	18	2.6

**Table 2:** Antioxidant activities (mg·mL^−1^) of peel and pulp in AFIs.

	Peel	Pulp
DPPH (IC_50_)	0.124 ± 0.009	0.237 ± 0.017 ***
ABTS (IC_50_)	0.130 ± 0.008	0.211 ± 0.010 ***

Results are expressed as means ± standard deviations (analyses from three replicates of 12 fruits per group). Means in the same line with the asterisks are significantly different (*** *p* < 0.001). The DPPH and ABTS IC_50_ values of V_C_ were 0.007 mg·mL^−1^ and 0.012 mg·mL^−1^, respectively.

## Data Availability

The data that support the findings of this study are available in the Supplementary Materials of this article.

## Supplementary Materials

The following supporting information can be downloaded at https://www.mdpi.com/article/10.3390/molecules29081733/s1, Figure S1: Total ion current of one quality control sample by mass spectrometry detection (A,B) and multi-peak detection plot of metabolites in the multiple reaction monitoring mode (C,D). A and C were acquired in positive ionization mode. B and D were acquired in negative ionization mode. The abscissa is the retention time of the metabolite and the ordinate is the ionic strength of the ion. Each different color mass spectrum peak represents a detected metabolite; Figure S2: Total ions current overlaps of the three quality control samples by mass spectrometry detection. A, TIC overlay plot in positive ionization mode. B, TIC overlay plot in negative ionization mode. The abscissa is the retention time of the metabolite and the ordinate is the ionic strength of the ion.; Table S1: Metabolite information of peel and pulp; Table S2: List of differential metabolites between peel and pulp; Table S3: Pathway annotation and enrichment analysis of key differential metabolites using KEGG pathway database; Table S4: List of significant pathways.

## References

[1] Singh B., Singh J.P., Kaur A., Singh N. (2020). Phenolic Composition, Antioxidant Potential and Health Benefits of Citrus Peel. Food Res. Int..

[2] Jabri Karoui I., Marzouk B. (2013). Characterization of Bioactive Compounds in Tunisian Bitter Orange (*Citrus aurantium* L.) Peel and Juice and Determination of Their Antioxidant Activities. Biomed Res. Int..

[3] Lu X.M., Zhao C.Y., Shi H., Liao Y.C., Xu F., Du H.J., Xiao H., Zheng J.K. (2023). Nutrients and Bioactives in Citrus Fruits: Different Citrus Varieties, Fruit Parts, and Growth Stages. Crit. Rev. Food Sci. Nutr..

[4] Li P., Zeng S.L., Duan L., Ma X.D., Dou L.L., Wang L.J., Li P., Bi Z.M., Liu E.H.P. (2016). Comparison of Aurantii Fructus Immaturus and Aurantii Fructus Based on Multiple Chromatographic Analysis and Chemometrics Methods. J. Chromatogr. A.

[5] Li J.M., Luo Y., Zhan L.L., Gu Y.Z., Zhang W.G., Wen Q., Feng Y.L., Tan J. (2022). Comprehensive Chemical Profiling of the Flowers of *Citrus aurantium* L. var. amara Engl. and Uncovering the Active Ingredients of Lipid Lowering. J. Pharm. Biomed..

[6] Suntar I., Khan H., Patel S., Celano R., Rastrelli L. (2018). An Overview on *Citrus aurantium* L.: Its Functions as Food Ingredient and Therapeutic Agent. Oxid. Med. Cell. Longev..

[7] Lee S.H., Yumnam S., Hong G.E., Raha S., Venkatarame Gowda Saralamma V., Lee H.J., Heo J.D., Lee S.J., Lee W.S., Kim E.H. (2015). Flavonoids of Korean *Citrus aurantium* L. Induce Apoptosis via Intrinsic Pathway in Human Hepatoblastoma HepG2 Cells: *Citrus* Flavonoids Induce Apoptosis in HepG2 Cells. Phytother. Res..

[8] Wolffenbüttel A.N., Zamboni A., Becker G., Dos Santos M.K., Borille B.T., De Cássia Mariotti K., Fagundes A.C., De Oliveira Salomón J.L., Coelho V.R., Ruiz L.V. (2018). *Citrus* Essential Oils Inhalation by Mice: Behavioral Testing, GCMS Plasma Analysis, Corticosterone, and Melatonin Levels Evaluation. Phytother. Res..

[9] Shen C.Y., Wan L., Wang T.X., Jiang J.G. (2019). *Citrus aurantium* L. var. amara Engl. Inhibited Lipid Accumulation in 3T3-L1 Cells and Caenorhabditis Elegans and Prevented Obesity in High-Fat Diet-Fed Mice. Pharmacol. Res..

[10] Shen C.Y., Wang T.X., Jiang J.G., Huang C.L., Zhu W. (2020). Bergaptol from Blossoms of *Citrus aurantium* L. var. amara Engl Inhibits LPS-Induced Inflammatory Responses and Ox-LDL-Induced Lipid Deposition. Food Funct..

[11] Oliveira S.A.C., Zambrana J.R.M., Di Iorio F.B.R., Pereira C.A., Jorge A.O.C. (2013). The Antimicrobial Effects of *Citrus limonum* and *Citrus aurantium* Essential Oils on Multi-Species Biofilms. Braz. Oral Res..

[12] Shen C.Y., Wang T.X., Zhang X.M., Jiang J.G. (2017). Various Antioxidant Effects Were Attributed to Different Components in the Dried Blossoms of *Citrus aurantium* L. var. amara Engl. J. Agric. Food Chem..

[13] Jia S., Hu Y., Zhang W., Zhao X., Chen Y., Sun C., Li X., Chen K. (2015). Hypoglycemic and Hypolipidemic Effects of Neohesperidin Derived from *Citrus aurantium* L. in Diabetic KK-Ay Mice. Food Funct..

[14] Hamdan D.I., Mahmoud M.F., Wink M., El-Shazly A.M. (2014). Effect of Hesperidin and Neohesperidin from Bittersweet Orange (*Citrus aurantium* var. bigaradia) Peel on Indomethacin-Induced Peptic Ulcers in Rats. Environ. Toxicol. Pharmacol..

[15] Liang Z., Sham T., Yang G., Yi L., Chen H., Zhao Z. (2013). Profiling of Secondary Metabolites in Tissues from *Rheum palmatum* L. Using Laser Microdissection and Liquid Chromatography Mass Spectrometry. Anal. Bioanal. Chem..

[16] Yi L., Liang Z.T., Peng Y., Yao X., Chen H.B., Zhao Z.Z. (2012). Tissue-Specific Metabolite Profiling of Alkaloids in Sinomenii Caulis Using Laser Microdissection and Liquid Chromatography–Quadrupole/Time of Flight-Mass Spectrometry. J. Chromatogr. A.

[17] Chang X., Li J., Ju M., Yu H., Zha L., Peng H., Wang J., Peng D., Gui S. (2021). Untargeted Metabolomics Approach Reveals the Tissue-Specific Markers of Balloon Flower Root (Platycodi Radix) Using UPLC-Q-TOF/MS. Microchem. J..

[18] Witzell J., Gref R., Näsholm T. (2003). Plant-Part Specific and Temporal Variation in Phenolic Compounds of Boreal Bilberry (*Vaccinium myrtillus*) Plants. Biochem. Syst. Ecol..

[19] Castro-Alves V., Kalbina I., Nilsen A., Aronsson M., Rosenqvist E., Jansen M.A.K., Qian M., Öström Å., Hyötyläinen T., Strid Å. (2021). Integration of Non-Target Metabolomics and Sensory Analysis Unravels Vegetable Plant Metabolite Signatures Associated with Sensory Quality: A Case Study Using Dill (*Anethum graveolens*). Food Chem..

[20] Zhao Y., Chu S., Gui S., Qin Y., Xu R., Shan T., Peng H. (2021). Tissue-Specific Metabolite Profiling of *Fallopia multiflora* (*Heshouwu*) and *Fallopia multiflora* var. *angulata* by Mass Spectrometry Imaging and Laser Microdissection Combined with UPLC-Q/TOF-MS. J. Pharm. Biomed..

[21] Li W., Wen L.C., Chen Z.C., Zhang Z.L., Pang X.L., Deng Z.C., Liu T., Guo Y.F. (2021). Study on Metabolic Variation in Whole Grains of Four Proso Millet Varieties Reveals Metabolites Important for Antioxidant Properties and Quality Traits. Food Chem..

[22] Gui A.H., Gao S.W., Zheng P.C., Feng Z.H., Liu P.P., Ye F., Wang S.P., Xue J.J., Xiang J., Ni D.J. (2023). Dynamic Changes in Non-Volatile Components during Steamed Green Tea Manufacturing Based on Widely Targeted Metabolomic Analysis. Foods.

[23] Wang Z.H., Gan S., Sun W.J., Chen Z.D. (2022). Widely Targeted Metabolomics Analysis Reveals the Differences of Nonvolatile Compounds in Oolong Tea in Different Production Areas. Foods.

[24] Ma Y.B., Li J.H., Li J.L., Yang L., Wu G.L., Liu S.Y. (2022). Comparative Metabolomics Study of Chaenomeles speciosa (Sweet) Nakai from Different Geographical Regions. Foods.

[25] Liao Z., Liu X., Zheng J., Zhao C., Wang D., Xu Y., Sun C. (2023). A Multifunctional True Caffeoyl Coenzyme A *O*-MethyltransFerase Enzyme Participates in the Biosynthesis of Polymethoxylated Flavones in Citrus. Plant Physiol..

[26] Zhu C., Zhou X., Long C., Du Y., Li J., Yue J., Pan S. (2020). Variations of Flavonoid Composition and Antioxidant Properties among Different Cultivars, Fruit Tissues and Developmental Stages of Citrus Fruits. Chem. Biodivers..

[27] Shi J.Y., Cai W.J., Lin W.D., Zhang S., Luo R. (2021). Comparison between Peel and Pulp of Aurantii Fructus Immaturus by UPLC Fingerprint and Multicomponent Quantitative Analysis. China J. Chin. Mater. Med..

[28] Moulehi I., Bourgou S., Ourghemmi I., Tounsi M.S. (2012). Variety and Ripening Impact on Phenolic Composition and Antioxidant Activity of Mandarin (*Citrus reticulate Blanco*) and Bitter Orange (*Citrus aurantium* L.) Seeds Extracts. Ind. Crop. Prod..

[29] Chen H.F., Zhang W.G., Yuan J.B., Li Y.G., Yang S.L., Yang W.L. (2012). Simultaneous Quantification of Polymethoxylated Flavones and Coumarins in *Fructus aurantii* and *Fructus aurantii Immaturus* using HPLC–ESI-MS/MS. J. Pharm. Biomed..

[30] Khan F.A., Maalik A., Murtaza G. (2016). Inhibitory Mechanism Against Oxidative Stress of Caffeic Acid. J. Food Drug Anal..

[31] Mancuso C., Santangelo R. (2014). Ferulic Acid: Pharmacological and Toxicological Aspects. Food Chem. Toxicol..

[32] Zhu M., Tang X., Zhu Z., Gong Z., Tang W., Hu Y., Cheng C., Wang H., Sarwar A., Chen Y. (2023). STING Activation in Macrophages by Vanillic Acid Exhibits Antineoplastic Potential. Biochem. Pharmacol..

[33] Ooghe W.C., Ooghe S.J., Detavernier C.M., Huyghebaert A. (1994). Characterization of Orange Juice (*Citrus sinensis*) by Polymethoxylated Flavones. J. Agric. Food Chem..

[34] Gao Z., Gao W., Zeng S.L., Li P., Liu E.H. (2018). Chemical Structures, Bioactivities and Molecular Mechanisms of Citrus Polymethoxyflavones. J. Funct. Foods.

[35] De Moraes Barros H.R., De Castro Ferreira T.A.P., Inés Genovese M. (2012). Antioxidant Capacity and Mineral Content of Pulp and Peel from Commercial Cultivars of Citrus from Brazil. Food Chem..

[36] Zeng S.L., Li S.Z., Xiao P.T., Cai Y.Y., Chu C., Chen B.Z., Li P., Li J., Liu E.H. (2020). Citrus Polymethoxyflavones Attenuate Metabolic Syndrome by Regulating Gut Microbiome and Amino Acid Metabolism. Sci. Adv..

[37] Huang R., Zhou Y., Jin F., Zhang J., Ji F., Bai Y., Pei D. (2022). Metabolome and Transcriptome Profiling Unveil the Mechanisms of Polyphenol Synthesis in the Developing Endopleura of Walnut (*Juglans regia* L.). Int. J. Mol. Sci..

[38] Chen W., Gong L., Guo Z., Wang W., Zhang H., Liu X., Yu S., Xiong L., Luo J. (2013). A Novel Integrated Method for Large-Scale Detection, Identification, and Quantification of Widely Targeted Metabolites: Application in the Study of Rice Metabolomics. Mol. Plant.

[39] Qu X.J., Hu S.Q., Li T., Zhang J.Q., Wang B.S., Liu C.L. (2022). Metabolomics Analysis Reveals the Differences between Bupleurum chinense DC. and Bupleurum scorzonerifolium Willd. Front. Plant Sci..

[40] Yang M., Yin M.Z., Chu S.S., Zhao Y.J., Fang Q.Y., Cheng M.E., Peng H.S., Huang L.Q. (2022). Colour, Chemical Compounds, and Antioxidant Capacity of Astragali Radix based on Untargeted Metabolomics and Targeted Quantification. Phytochem. Anal..

[41] Litewski S., Koss-Mikołajczyk I., Kusznierewicz B. (2024). Comparative Analysis of Phytochemical Profiles and Selected Biological Activities of Various Morphological Parts of Ligustrum vulgare. Molecules.

[42] Sarega N., Imam M.U., Ooi D.J., Chan K.W., Md Esa N., Zawawi N., Ismail M. (2016). Phenolic Rich Extract from *Clinacanthus nutans* Attenuates Hyperlipidemia-Associated Oxidative Stress in Rats. Oxid. Med. Cell. Longev..

