# Supplementary material for: Widely Targeted Metabolomics Analysis Reveals Metabolites Important for Antioxidant Properties and Quality Traits in Different Fruit Parts of Aurantii Fructus Immatures
Source: Molecules. 2024 Apr 11;29(8):1733. doi: 10.3390/molecules29081733 (PMC11051935; doi:10.3390/molecules29081733)
Supplement: Supplementary file 1 [file molecules-29-01733-s001.zip › Supplementary Figure.pdf]

Supplementary Figure

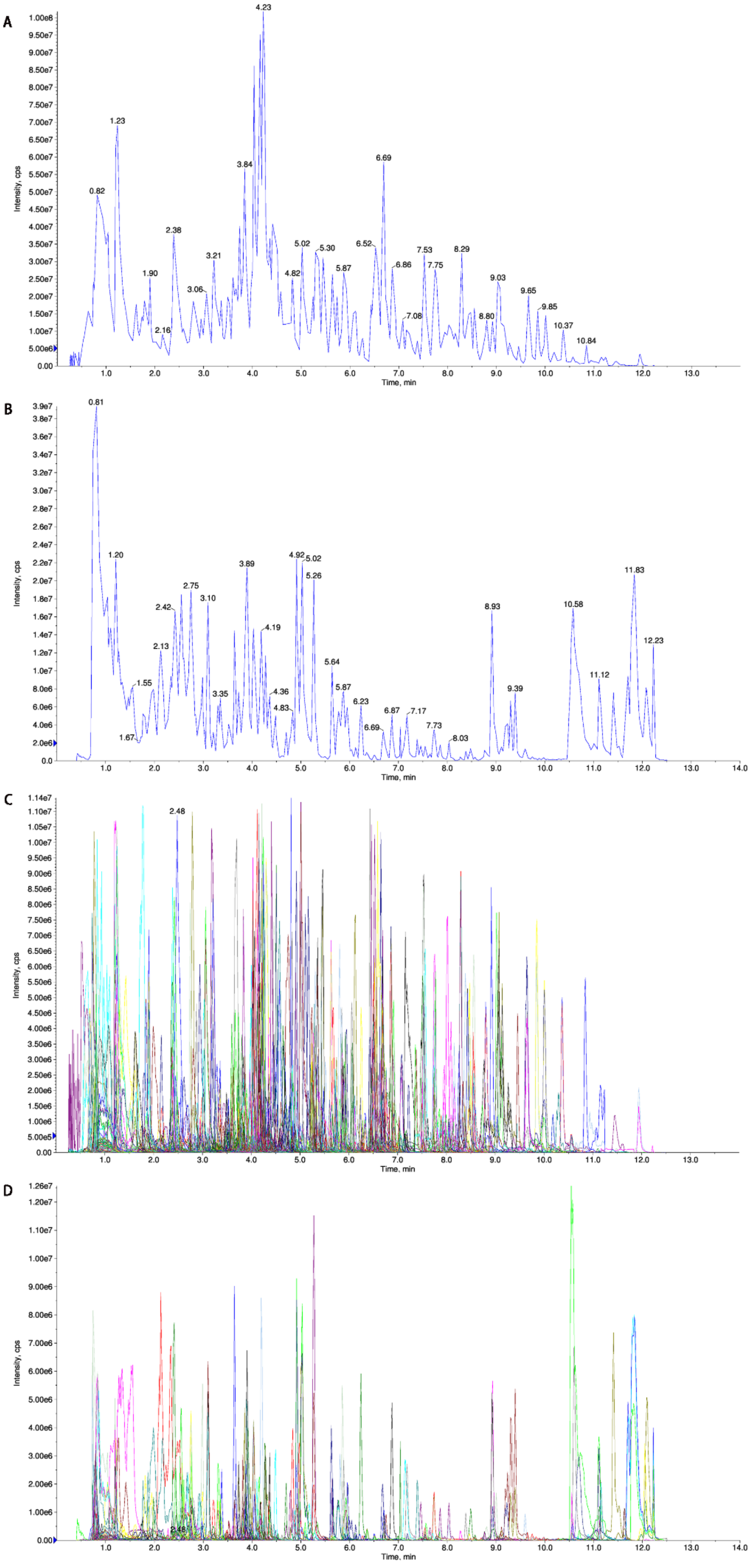

Fig. S1. Total ion current of one quality control sample by mass spectrometry detection (**A**, **B**) and multi-peak detection plot of metabolites in the multiple reaction monitoring mode (**C**, **D**). **A** and **C** were acquired in positive ionization mode. **B** and **D** were acquired in negative ionization mode. The abscissa is the retention time of the metabolite and the ordinate is the ionic strength of the ion. Each different color mass spectrum peak represents a detected metabolite.

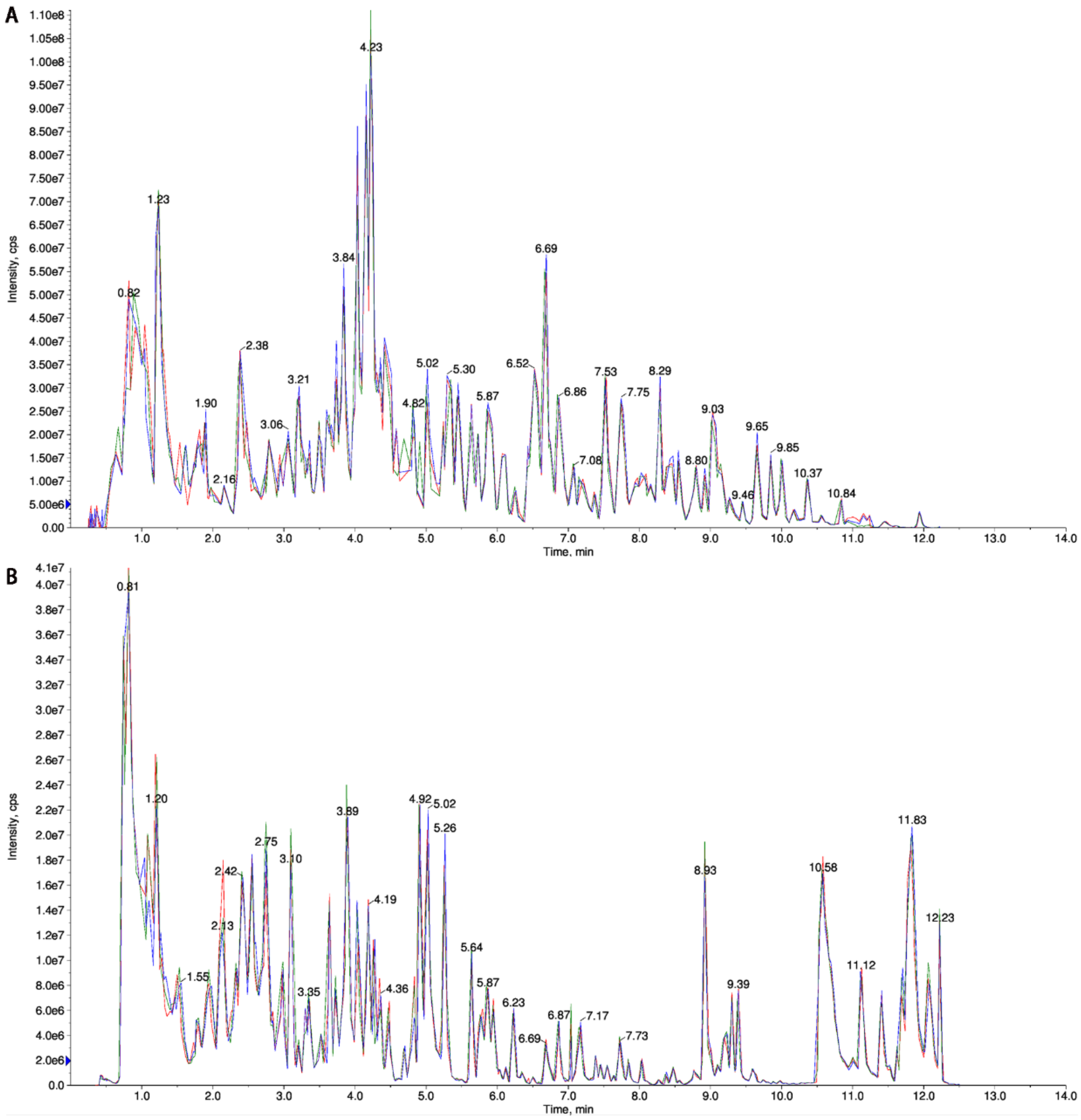

Fig. S2. Total ions current overlaps of the three quality control samples by mass spectrometry detection. **A**, TIC overlay plot in positive ionization mode. **B**, TIC overlay

plot in negative ionization mode. The abscissa is the retention time of the metabolite and the ordinate is the ionic strength of the ion.
